# Pregnant women’s acceptability of intermittent preventive treatment with dihydroartemisinin-piperaquine from user and provider’s perspectives: qualitative findings from the pilot implementation in Papua, Indonesia

**DOI:** 10.1186/s12884-026-09036-x

**Published:** 2026-04-06

**Authors:** Jenna Hoyt, Freis Candrawati, Firdaus Hafidz, Asal Wahyuni Erlin Mulyadi, Enny Kenangalem, Ida Safitri Laksanawati, Reynold Ubra, Jeanne Rini Poespoprodjo, Jenny Hill

**Affiliations:** 1 Department of Clinical Sciences, Liverpool School of Tropical Medicine, Liverpool, UK; 2 Department of Health Policy and Management, Faculty of Medicine, Public Health and Nursing, Universitas Gadjah Mada, Yogyakarta, Indonesia; 3 Timika Research Facility, Papuan Health and Community Development Foundation, Mimika, Papua, Indonesia; 4 Centre for Child Health-PRO, Faculty of Medicine, Public Health and Nursing, Universitas Gadjah Mada, Yogyakarta, Indonesia; 5 Mimika District Health Office, Mimika, Papua, Indonesia

**Keywords:** Malaria in pregnancy, IPTp-DP, Acceptability, Implementation research, Indonesia, Primary care

## Abstract

**Introduction:**

Intermittent preventive treatment with dihyroartemisinin-piperaquine (IPTp-DP) was found to be safe and more cost-effective than the current policy of single screening and treatment for the prevention of malaria in pregnancy in Papua, Indonesia. However, acceptability of the strategy under routine conditions has yet to be explored. This qualitative study explored pregnant women’s acceptability of IPTp-DP in the context of a Ministry of Health pilot programme.

**Methods:**

In-depth interviews with 94 pregnant women, 103 healthcare providers/managers and 13 husbands were conducted at two time points in ten health centres as part of a wider mixed method evaluation of implementation feasibility. Data were coded using Sekhon’s Acceptability constructs. Thematic framework analysis was used to explore women’s acceptability of IPTp-DP over time and identify potential drivers of change, which were then triangulated with data from healthcare providers/managers and husbands.

**Results:**

Women perceived malaria in pregnancy to be a serious issue and valued the opportunity to prevent malaria with IPTp-DP. They had initial concerns about the safety of taking DP in pregnancy without a diagnostic test. Concerns about safety and side effects were allayed with information and reassurance from healthcare providers. Changes in women’s acceptability of IPTp-DP were mainly driven by improved communication about the intervention from providers and enhanced engagement with family members/husbands through quality improvement activities.

**Conclusions:**

Health information about the benefits and safety of IPTp-DP for preventing malaria in pregnancy, together with support from spouses, were important drivers of women's acceptability of, and adherence to, IPTp-DP in this setting.

**Supplementary Information:**

The online version contains supplementary material available at 10.1186/s12884-026-09036-x.

## Introduction

Malaria infection in pregnancy is linked to adverse outcomes for mother and baby, including severe maternal anaemia, low birth weight, preterm delivery and stillbirth [1]. Malaria in pregnancy represents a significant public health challenge in South-East Asia, with over 52 million pregnancies at risk [2] and no regional strategy for prevention. In Papua, Indonesia, the situation is complicated by the presence of both *Plasmodium vivax* and *Plasmodium falciparum*, high endemicity resulting in asymptomatic infections and sulphadoxine-pyrimethamine (SP) resistance [3, 4] As a result, intermittent preventive treatment (IPTp) with SP is not recommended. In 2012, Indonesia became the first country in the region to implement the single screen and treat (SST) strategy for the prevention of malaria in pregnancy, alongside passive case detection and the use of long-lasting insecticide-treated nets [5]. SST involves testing pregnant women for malaria at their first antenatal care (ANC) visit and administering treatment if positive. A recent clinical trial found that IPTp with dihydroartemisinin-piperaquine (DP) is a safe, efficacious and cost-effective option for the prevention of malaria in pregnancy in Papua, Indonesia, a setting with moderate to high malaria transmission and high levels of SP resistance [6, 7]. To inform a policy shift, evidence on women’s acceptability to IPTp-DP is urgently needed.

Acceptability of IPTp-DP among pregnant women and healthcare providers was previously explored in the context of a clinical trial in Indonesia [8]. Providers raised concerns about administering antimalarials presumptively, without a diagnostic test, but women appreciated the opportunity to prevent malaria [8].Despite these encouraging findings, adherence to the 3-day DP regimen and unpleasant side effects remain potential barriers to optimal implementation of IPTp-DP in sub-Saharan Africa and Southeast Asia [8–11]. A recent study in Kenya found that adherence to IPTp-DP was enhanced when delivered alongside targeted information on side effects and how to minimise them [10, 11]. Similarly in Indonesia, healthcare providers highlighted the need for effective communication prior to a shift in policy from screening and treatment to preventive treatment [8]. Implementation research exploring acceptability to IPTp-DP in routine settings is needed to identify barriers and inform mitigation strategies.

This qualitative study was part of a mixed methods evaluation of the feasibility of a Ministry of Health (MoH) pilot of IPTp-DP delivered through routine ANC services in a high malaria burden setting in Papua Indonesia. The purpose of this research was to explore changes in women’s acceptability towards IPTp-DP over time, identify potential drivers of change, and provide key insights for national scale-up.

## Methods

### Study design & context

We conducted a qualitative study in the context of a pilot implementation of IPTp-DP to explore pregnant women’s perceptions and attitudes towards IPTp-DP and how they changed over time. Perspectives from healthcare providers, managers and husbands were used to support, contrast or expand on women’s views towards IPTp-DP. Thematic analysis was guided by Sekhon’s acceptability framework.

The IPT-DP pilot was implemented in ten community health centres (6 semi-urban, 4 urban) and ran from February 2022 to November 2023, in Mimika District in Papua Province, Indonesia. There was a break in IPTp-DP delivery during a nationwide stock out of DP, from May to September 2022, which led to the pilot being temporarily halted. The impact of the stock out on coverage is discussed elsewhere [12]. The findings of this study are reported in accordance with the Standards for Reporting Qualitative Research (SRQR) guidelines (Additional file 1)**.**

Mimika district is comprised of low and highlands with an estimated population of 313,016, with business and commerce being the principal economic activities [13]. Malaria is endemic with cases predominately in the lowlands. Among pregnant women at delivery, parasitaemia prevalence is 16.8% but fewer than half of these infections are associated with fever (35%) [4]. ANC services in Mimika District are delivered at 26 community health centres (*puskesmas*), and via a network of auxiliary health centres (*posyandu*) where community integrated services are held monthly or bi-monthly in villages. The pilot was conducted in health centres and their auxiliary and community services that were accessible by road all year round. Most pregnant women in Mimika attend ANC at a health facility at least once during their pregnancy (97%), however less than half make at least four ANC visits (46%) (Mimika district health office 2019). Since 2020, women are recommended to make six ANC visits during their pregnancy [14]. DP is the recommended first line treatment for uncomplicated malaria in pregnant women in all trimesters, including pregnant women who test positive during SST at their first ANC visit.

### IPTp-DP pilot implementation

Prior to implementation, healthcare providers from pilot facilities received training on IPTp-DP by the study team. Pregnant women attending monthly ANC visits in the second and third trimester of pregnancy were offered IPTp-DP (3 tablets of 40 mg dihydroartemisinin and 320 mg piperaquine per day for three days, i.e. 9 tablets). The first dose of DP was administered by directly observed therapy (DOT) with subsequent doses provided for free to the women to be taken at home. Follow up home visits or phone calls from healthcare providers were conducted to support adherence. In addition, promotional materials (job aids, leaflets, videos) developed and distributed by the Ministry of Health were made available in pilot facilities. IPTp-DP was added to the Continuous Quality Improvement (CQI) programme. CQI training on IPTp-DP was provided for all facility staff and district managers at the start of the pilot and a CQI lead was identified at each facility. In the initial workshop, participants worked in facility-based groups to identify bottlenecks in IPTp-DP delivery, set performance targets, and develop action plans. Each health centre presented their plans and received feedback from peers and facilitators. In subsequent CQI workshops, progress was reviewed against key performance indicators (IPTp-DP coverage, adherence, communication quality, and reporting completeness), and participants discussed challenges such as drug stock-outs or side-effect counselling and updated the action plans. The workshops also included refresher sessions on effective communication with pregnant women and their families, data use for quality improvement, and community engagement strategies. Further details on the four CQI sessions and outcomes are published in the main evaluation paper [12].

### Study participants & data collection

**Fig. 1:**
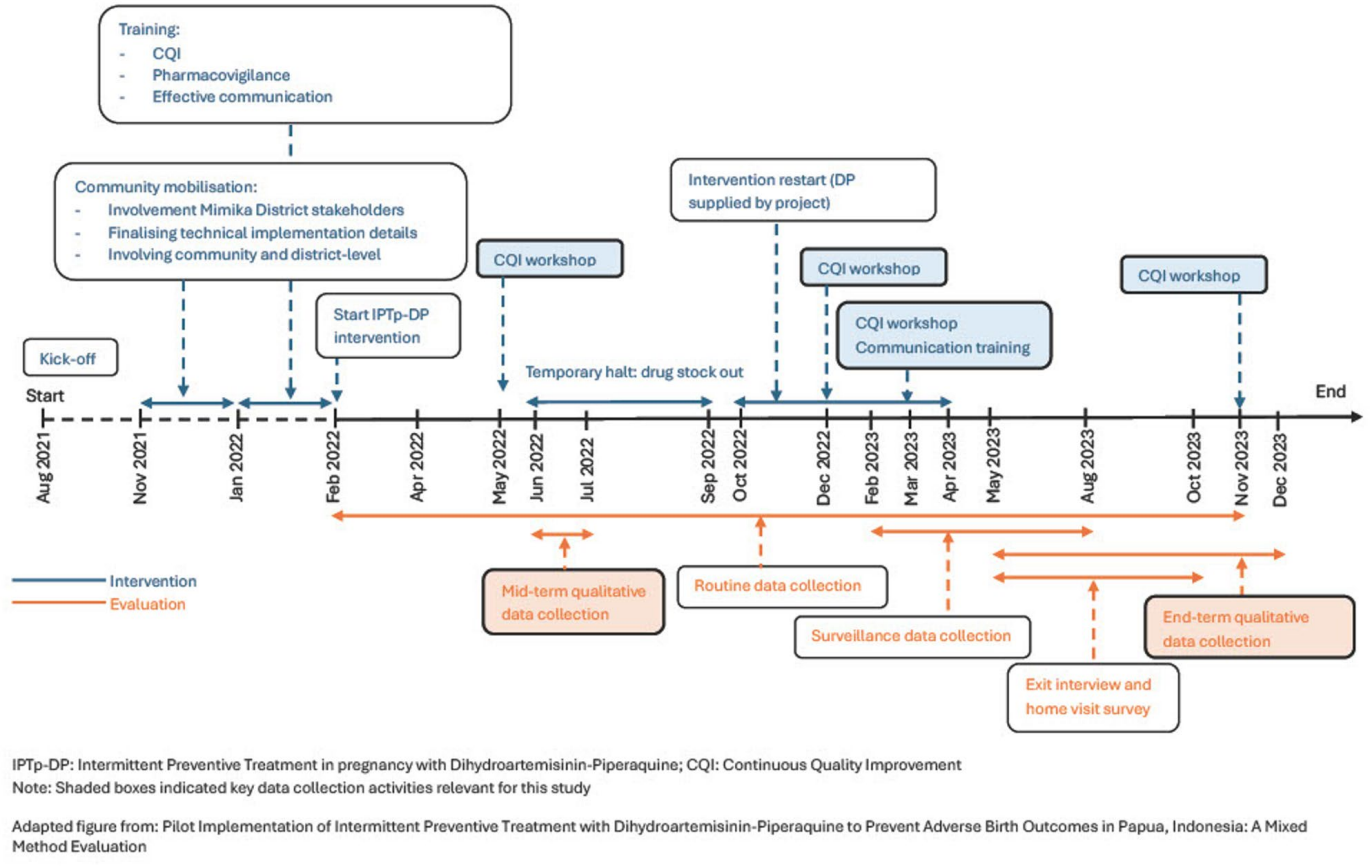
Timeline for IPTp-DP Pilot Implementation and Evaluation in Mimika District

Qualitative data was collected at two time points (Fig. 1). Midline data was collected between June - July 2022, which coincided with a national stock out of DP. Endline data was collected between June – December 2023. At both time points, in-depth interviews (IDIs) were conducted with pregnant women, healthcare providers and managers. Pregnant women in the 2nd or 3rd trimester, aged 15–49 years old, HIV uninfected and attending routine ANC visits were eligible. Three to five women were purposively selected from each of the 10 community health centres to ensure inclusion of primi- and multi-gravidae women and a range of ANC visits (i.e. women attending first visit versus subsequent visits). Healthcare providers delivering ANC services (at both health centres and posts) were purposively selected from each community health centres in the pilot. Health managers were purposively selected from the district level (both mid- and endline), provincial and central levels (endline only), including from maternal and child health, and malaria focal points. Additional endline data was collected using IDIs with husbands, who were purposively sampled from a list of IPTp-DP eligible pregnant women in their 2nd or 3rd trimester at the health facilities, to include a mix of first-time fathers and those with previous children, residing in the catchment areas of the pilot facilities.

Topic guides for IDIs with pregnant women assessed: (1) their acceptability of IPTp-DP compared to SST, (2) their perceptions of being given preventive treatment without a diagnostic test, (3) any challenges faced with access and adherence to IPTp-DP. The topic guides for IDIs with healthcare providers and managers explored: (1) their acceptability of IPTp-DP, (2) any adaptations to their work practices required to implement IPTp-DP, (3) their perceptions of the feasibility of implementing the intervention at scale (including resource constraints), and (4) their recommendations on factors to ensure effective implementation. Themes that emerged from midline data collection were used to refine and expand the guides used at endline data collection. The topics guides for IDIs with husbands explored: (1) their perceptions of malaria in pregnancy risk / benefits of prevention, (2) their acceptability of IPTp-DP, (3) their willingness to support their wives to take IPTp (Additional file 2).

Interviews were conducted in Indonesian by five members of the research team (four females and one male), who hold either post-graduate degrees in health sciences (field supervisors) or bachelor’s degrees in physiology. All members of the team received training in qualitative methods, data collection techniques and good clinical practices. Interviews were audio recorded, transcribed verbatim and translated to English. To ensure confidentiality and anonymity of the participants, audio files and transcripts were pseudonymised and labelled with a unique ID number and accessed only by authorised members of the research team.

### Data analysis

Transcripts were imported into Nvivo 1.7 (QSR international) and coded thematically by one researcher (JHo) using both deductive and inductive coding. Separate a priori coding structures for pregnant women and healthcare providers/managers were developed to guide coding (Additional file 3), with emergent themes and sub-themes added and then refined as the coding progressed. Pregnant women transcripts were coded using constructs from two published frameworks: (1) access framework (acceptability, accessibility, accommodation, affordability, availability) [15] and (2) Sekhon’s acceptability framework (affective attitude, burden, ethicality, intervention coherence, opportunity costs, perceived effectiveness and self-efficacy) [16]. The acceptability construct definitions were adapted to reflect the intervention **(**Table 1). In addition, the coding structure included context (malaria knowledge & experiences, ANC experiences) and adherence to IPTp-DP. Healthcare provider and manager data was coded thematically across the health system building blocks (governance, finance, human resources, health information, products & technology and service delivery). ^17^ Transcripts from husbands (endline only) were coded using a simplified structure that comprised (1) knowledge of IPTp-DP, (2) experiences of IPTp-DP and (3) opinions of IPTp-DP (perceived benefits and concerns). Coding validation discussions were held with the field team to contextualise the data and ensure the meanings were interpreted appropriately.

**Table 1:** Acceptability framework constructs and adapted definitions

Construct	Definition adapted to IPTp-DP
Affective attitude	How women feel about IPTp-DP, including taking antimalarials in pregnancy
Intervention coherence	What women understand about how IPTp-DP works and why it is recommended that they take it
Burden	The effort involved in taking IPTp-DP, including accessing IPTp-DP and dealing with side effects
Self-efficacy	The degree to which women feel they can take IPTp-DP, complete the doses at home and manage side effects
Ethicality	Why women feel IPTp-DP is important to them and the degree to which their decision is supported within their interpersonal networks
Perceived effectiveness	The degree to which women feel IPTp-DP protects them against malaria in pregnancy
Opportunity costs	What they need to give up in order to take IPTp-DP

Data analysis involved several steps. First, the pregnant women data was examined at both time points with themes mapped to the acceptability constructs to generate an overall picture of women’s attitudes towards IPTp-DP. Next, themes across the acceptability constructs were extracted into a matrix and examined across the two time points to elucidate changes in women’s perceptions towards IPTp-DP between midline and endline. Here, data that indicated changes in perceptions, and in what way they changed, were highlighted and potential drivers of these changes were identified and discussed among the research team. Finally, data from healthcare providers, managers and husbands were explored, extracted and added to the matrix if it supported the findings or provided divergent views. This process of triangulation helped expand and strengthen the interpretation of what influenced pregnant women’s acceptability of IPTp-DP.

**Fig. 2:**
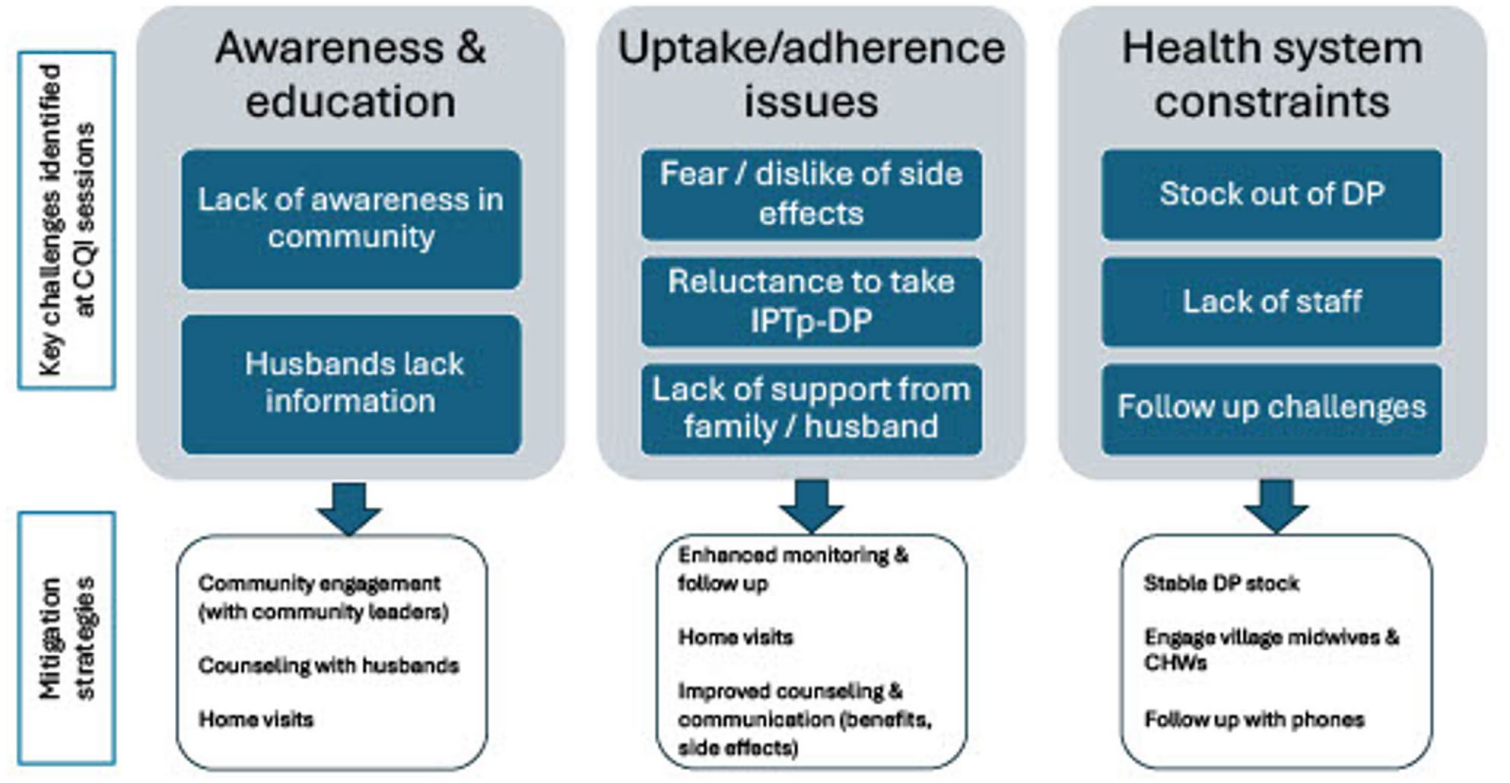
Key challenges and mitigation strategies extracted form CQI workshop reports

The outcomes from the four CQI workshops were examined and extracted to provide context for the analysis. Data was extracted on (1) key challenges identified across the ten facilities and (2) strategies put in place by the facilities to mitigate the challenges (Fig. 2). Relevant contextual information was used to help explain the qualitative data, to understand the corrective actions taken as a result of CQI to improve healthcare provider and facility practices, and how these actions may have influenced women’s perceptions of IPTp-DP.

### Ethics

The study received ethical approval from the Liverpool School of Tropical Medicine (#21–054) and the Universitas Gadjah Mada Research Ethics Committees (#1198). All study participants provided written informed consent.

## Results

A total of 94 IDIs with pregnant women (47 midline, 47 endline), 69 healthcare providers (29 midline, 40 endline) and 31 health managers (14 midline, 17 endline) were conducted across ten community health facilities in Mimika District. Health managers included heads of community health centres *(puskesmas)* and district focal points for malaria, maternal and child health, and family health. In addition, three malaria and family health programme managers at the provincial and national level were interviewed at endline. Finally, 13 husbands were interviewed at endline. The characteristics of pregnant women are presented in Table 2 and for healthcare providers (Additional File 4). Key findings presented include pregnant women’s acceptability of IPTp-DP, changes in perceptions over time and what drove those changes Table 3.

**Table 2:** Characteristics of pregnant women at midline and endline

Pregnant women	Midline (*n* = 47)	Endline (*n* = 47)
Marital status		
* Married*	41 (87%)	37 (79%)
* Single/divorced/widow*	6 (13%)	10 (21%)
Age range		
* 15–20*	7 (15%)	12 (26%)
* 21–34*	33 (70%)	31 (66%)
* >=35*	7 (15%)	4 (8%)
Education level		
* No education/primary*	8 (17%)	4 (8%)
* Middle / high school*	29 (62%)	36 (77%)
* Diploma / university*	10 (21%)	7 (15%)
Religion		
* Catholic / Protestant*	28 (60%)	34 (72%)
* Islam / other*	19 (40%)	13 (28%)
Region		
* Urban*	22 (47%)	20 (43%)
* Semi-urban*	25 (53%)	27 (57%)
Gestational age		
* Second trimester*	9 (19%)	15 (32%)
* Third trimester*	38 (81%)	32 (68%)
Gravidity		
* Primigravidae*	8 (17%)	18 (38%)
* Multigravidae*	39 (83%)	29 (62%)
ANC visits		
* 3 or less times*	11 (23%)	19 (40%)
* > 3 times*	36 (77%)	28 (60%)
Number of IPTp-DP doses		
* 0 doses*	5 (11%)	6 (13%)
* 1 dose*	20 (42%)	14 (30%)
* 2 doses*	6 (13%)	9 (19%)
* 3 doses*	8 (17%)	11 (23%)
* 4 + doses*	8 (17%)	7 (15%)
Ever experienced malaria		
* Yes*	38 (81%)	36 (77%)
* No*	9 (19%)	11 (23%)

**Table 3:** Pregnant women’ acceptability of IPTp over time and drivers of change

Construct	Key acceptability themes	Changes in acceptability	Drivers of change
Affective attitude	• Women want to prevent malaria • Concerns about taking drugs in pregnancy	Reduced concerns about side effects and negative impact of DP on fetus improved women’s attitude towards taking drugs in pregnancy	Effective messaging from health providers about the safety of DP CQI workshop on effective communication
Intervention coherence	• Understand IPTp-DP will prevent malaria infections • Uncertainty about why you would take drugs when not ill	Women’s understanding of the risks of malaria in pregnancy and the benefits of IPTp-DP improved	Effective communication from health providers about the risks of malaria in pregnancy & the importance of prevention CQI workshop on effective communication
Burden	• Unpleasant side effects of DP (nausea, vomiting, fatigue, dizziness) • Women not always given information about side effects	Dislike/fear of the side effects remained constant	No change over time.
Self-efficacy	• Women try to manage side effects at home • Women do not always adhere to DP doses at home because of side effects	Women’s confidence/ability to manage side effects effectively at home improved	Advice from health providers improved management of side effects, whilst home visits from HWs reassured women and family members that they were being monitored CQI workshop on effective communication
Ethicality	• Need support from husbands/family members to take up IPTp-DP and/or complete the doses at home	Greater support from husbands (and family) to take up and adhere to IPTp-DP enhanced their willingness to take up IPTp-DP	Engagement with husbands and the community increased the support women received for taking & completing IPTp-DP CQI mitigation strategy of enhanced community engagement
Perceived effectiveness	• IPTp-DP prevents malaria during pregnancy • Women feel healthier after taking IPTp-DP	Women’s belief that IPTp-DP prevented malaria remained constant	No change over time.
Opportunity costs	• No opportunity costs related to IPTp-DP identified	No changes identified	No change over time.

IPTp-DP: intermittent preventive treatment with dihydroartemisinin-piperaquine, CQI: continuous quality improvement

### Pregnant women’s acceptability of IPTp-DP

Women valued the opportunity to prevent malaria, which they perceived to be a serious issue during pregnancy, with IPTp-DP. However, this was often tempered by concerns about taking antimalarial drugs, which many considered a ‘strong’ medication, during pregnancy and potential harm to the baby. Other complaints included the bad taste and smell of DP and the size of the tablets. Interestingly, some women who had previously experienced malaria in pregnancy were particularly appreciative of IPTp-DP and focused immediately on the potential benefits, noting that taking medication (and experiencing potential side effects) was better than suffering with malaria.

Understanding the importance of preventing malaria during pregnancy and the role of IPTp-DP for chemoprevention was crucial for women’s decision making about whether to ‘risk’ taking DP while pregnant. Importantly, women wanted reassurance that DP was safe to take during pregnancy, without which some women did not feel comfortable to take the medication when they weren’t ill. Other women reported feeling better after taking the course of DP or feeling healthier than in previous pregnancies.

Common side effects reported included nausea, fatigue, vomiting and dizziness. For some women these side effects were present on each day of the regimen whereas for others they lessened over time. Side effects were cited as the main reason for non-adherence to the 3-day regimen and/or for declining the next IPTp-DP course. Many women described how they managed the side effects at home primarily by resting and eating prior to taking DP. Not all women were given information about potential side effects or how to manage them by healthcare providers.

Side effects also raised concerns among family members, notably husbands, who in some cases instructed their wives to stop taking DP. Support from husbands and family members was important for women when making the decision to take up IPTp-DP and for adherence to the doses taken at home. Several women indicated that their husbands wanted reassurance and information from a healthcare provider before permitting them to take IPTp-DP.

**Table:** 

Panel 1. Illustrative quotes for pregnant women’s acceptability of IPTp-DP
*“So when I was offered it*,* I immediately agreed*,* without my husband’s approval. Because malaria traumatized me. So those who are not pregnant are already so severe*,* especially with the condition of pregnant women.”* Pregnant woman_8, midline, 30 years, 3 IPTp-DP doses, multigravida *“Because pregnant women may not be able to take malaria medicine. They don’t want to drink because the malaria medicine is strong.”* Pregnant woman_14, midline, 33 years, 1 IPTp-DP dose, multigravida
*“…From the health workers*,* it is better to explain more and make demonstrations about health and malaria prevention. Must explain how the medicine works*,* the side effects*,* the impact if you don’t take the medicine. We*,* pregnant women*,* may not know yet….”* Pregnant woman_18, midline, 23 years, 3 IPTp-DP doses, primigravida *“I prefer to take medicine only when we are sick (ed. SST) because if we are not sick and then given medicine*,* I feel worried because medicine is a chemical*,* I am afraid that there will be other side effects that we do not know about. Unless I’m sick.”* Pregnant woman _21, midline, 26 years, 2 IPTp-DP doses, multigravida *“The malaria prevention program (IPTp-DP) is better because in my previous pregnancy it was difficult to gain weight. In this pregnancy*,* since taking malaria prevention medicines*,* I have been gaining weight every month and I feel much healthier.”* Pregnant woman _24, midline, 23 years, 2 IPTp-DP doses, multigravida
*“I: What is the reason you did not continue taking the medicine again? R: The reason was because I felt weak. I: Was it because of the medicine’s side effects? R: Yes*,* and another reason my husband forbad me. My husband asked me to stop*,* so I didn’t take it anymore.”* Pregnant woman _22, midline, 31 years, 1 IPTp-DP dose, multigravida

### Changes in pregnant women’s acceptability over time

Changes in women’s acceptability towards IPTp-DP were noted with respect to *affective attitude*,* intervention coherence*,* self-efficacy* and *ethicality.* Women’s perceptions of the *burden* and *perceived effectiveness* of IPTp-DP remained constant over time. At both time points no *opportunity costs* were identified. Providers expressed concern that interruptions in the delivery of IPTp-DP due to nationwide stockouts of DP would undermine women’s trust in the programme. However, this was not directly substantiated by the women themselves.

Lack of information about IPTp-DP, specifically about the safety of DP in pregnancy, led to initial hesitation and refusals by some women. Information and reassurance from health providers that DP was safe to take during pregnancy addressed women’s concerns and enhanced their positive perceptions of the intervention *(affective attitude)*. Although women’s desire to prevent malaria was consistent at both timepoints, their understanding of the preventive role of IPTp-DP deepened over time with improved messaging from healthcare providers *(intervention coherence)*.

Unpleasant side effects of DP remained a challenge for some women throughout the pilot *(burden)*. However, information and reassurance from healthcare providers reduced women’s concerns about the side effects (i.e. what was normal to expect, did not represent harm to the baby) and equipped women with strategies to minimise them. Advice from providers on how to manage side effects helped women (and their families) deal effectively with side effects at home *(self-efficacy)*. In addition, home visits from providers comforted women as they felt that being monitoring relieved some of the worries around side effects.

Support from family members, husbands in particular, remained important for women’s decision making about IPTp-DP throughout the pilot implementation *(ethicality)*. Lack of spousal support was reported by healthcare providers as a barrier to women taking up IPTp-DP, despite in some cases the woman herself being willing. Most husbands appreciated the protection IPTp-DP afforded their wives and unborn children against malaria; however, they wanted to hear information from trusted sources (e.g. health providers) before supporting their wives to take IPTp-DP. As information about the importance and safety of IPTp-DP reached the community and households, some women received greater support for taking up IPTp-DP.

**Table:** 

Panel 2. Illustrative quotes for changes in pregnant women’s acceptability over time
*“The challenge is medicine…because it is difficult to build trust. We’ve worked hard to provide education to patients*,* but suddenly [DP] stopped in the middle of the road. It means that the trust that we have built in society has faded. Rebuilding an existing one is difficult.”* Healthcare provider_8, midline, female midwife with diploma
*“Yes*,* I was worried because I was afraid of side effects on the fetus*,* and malaria medicine is strong. However*,* after being explained by the midwife*,* my worries disappeared.”* Pregnant woman _40, endline, 27 years, 1 IPTp-DP dose, multigravida *“In April*,* the nurse explained it*,* but I didn’t quite understand what IPTp-DP was at that time. And since my husband was still away for work*,* we hadn’t discussed it yet. Then*,* because I had experienced malaria recently and knew how painful it could be*,* I decided to join the IPTp-DP program. Also*,* because the nurse explained it in detail*,* I understood it better.”* Pregnant woman _09, endline, 29 years, 1 IPTp-DP dose, primigravida *“In my opinion*,* first*,* those of us conducting these education sessions need to make sure that mothers understand and grasp the importance of malaria prevention. We need to raise their awareness. Without proper education*,* they might not understand*,* you know.”* Healthcare provider_19, endline, female midwife with bachelor’s degree
*“The important thing is we’ve received explanations from the midwives at the health center*,* and they’ve reminded us to eat a lot before taking the medicine to avoid feeling dizzy. In my experience*,* if I eat before taking the anti-malaria medicine*,* I don’t feel dizzy or weak.”* Pregnant woman _21, endline, 30 years, 3 IPTp-DP doses, multigravida *“I advise them to buy candies or consume foods/beverages with a pleasant taste to counteract the nausea*,* as there are no dietary restrictions.”* Health provider_22, endline, female pharmacist with bachelor’s degree *“Yes*,* pregnant women who felt nauseous and refused to take it again. However*,* health providers explain the benefits again*,* and even if they initially refuse*,* they eventually take it.”* Health provider_21, endline, female midwife with diploma *“I feel happy with how it’s currently implemented. It’s been going well because we’ve already been visited by health providers*,* which is different from the past.”* Husband_11, endline, 22 years, high school education, 1 child
*“Yes*,* I’m worried about the effects or side effects of IPTp-DP on pregnant women because I honestly haven’t received any data about the baby’s development*,* from slow thinking to delayed responses to things. That’s what worries me. I haven’t received direct official information from medical or health professionals*,* so this is based on my common understanding.”* Husband_04, endline, 45 years, bachelor’s degree education, no children *“Sometimes the family supports them*,* but sometimes not. We’ve encountered cases where the mother wanted to take it*,* but her husband didn’t allow it.”* Health provider_19, endline, female midwife with bachelor’s degree *“My husband is okay with it. He asked before. I explained it again because I had heard the explanation from the midwife. Eventually*,* he said*,* ‘Okay*,* it’s fine*,* it’s better.’”* Pregnant woman _02, endline, 28 years, 1 IPTp-DP dose, multigravida

### Key drivers of changes in women’s acceptability

Changes in women’s acceptability of IPTp-DP were largely driven by support from husbands and/or family members following improved communication and messaging by healthcare providers about the intervention and greater community engagement.

Healthcare providers described the impact of training on their communication practices, specifically how it improved their ability to connect with the women and convince them of the benefits of IPTp-DP. In addition, some providers felt that on-going education about the benefits of IPTp-DP helped persuade women to return for their next monthly course of IPTp-DP. Interestingly, the CQI trainings also improved healthcare providers’ understanding of IPTp-DP and alleviated their own concerns about presumptive treatment, giving them added confidence to reassure and convince women to take IPTp-DP.

During CQI sessions, lack of information about IPTp-DP among family members, specifically husbands, was raised as a key challenge. Mitigation strategies aimed at reaching the community and household level with information about IPTp-DP were developed. Home visits from providers emerged as a useful way to transmit information directly to family members. Community discussions were also conducted to reach a wide range of community members (e.g. religious leaders, village elders, men) with key information about IPTp-DP. Despite these activities, providers cautioned that concerns about the safety of DP persisted, a sentiment echoed by some husbands.

**Table:** 

Panel 3. Illustrative quotes for key drivers of changes in women’s acceptability
*“It [CQI training] was early last year*,* in February. Honestly*,* I myself found it a bit challenging. Healthy people being asked to take DP medication*,* an anti-malarial drug. They weren’t positive for malaria. But after attending that training*,* I became knowledgeable. I was truly convinced that it’s a government program. And after seeing the progress each month*,* it’s clear that it has a positive effect.”* Health provider_28, endline, female pharmacist with bachelor’s degree *“We learned about effective communication about [IPTp-DP]*,* how to approach and build rapport with pregnant women. We covered everything during the training in February 2023. The training was good*,* effective*,* and not boring. Continuous Quality Improvement (CQI) involves using data. Every time data from pregnant women is entered into the CQI system*,* we use it to create graphs and analyze trends.”* Health provider_18, endline, female midwife with diploma *“It depends on how the health providers educate the community. If the health providers effectively educate the community and it makes logical sense to them*,* they will accept it. Training for healthcare providers in interview techniques and education techniques is crucial. The success of an activity depends on how the providers educate the community.”* Health manager_14, endline, head of family health
*“According to what happened here*,* at first it was difficult to explain*,* because many of them did not accept it. Their reason was that they conveyed it to their husbands first*,* some of them asked permission from their husbands first*,* they said it to their families first*,* because this is a new program.”* Health provider_20, midline, female pharmacist with bachelor’s degree *“The best way is for healthcare providers to conduct home visits*,* so that pregnant women don’t have the option to refuse. Pregnant women should accept DHP malaria medication for prevention. Additionally*,* healthcare providers promote health awareness within patient families. Sometimes it’s challenging to provide education if the family is not receptive.”* Health provider_03, endline, female nurse with diploma *“Some are supportive*,* while others are not*,* from family members like husbands or mothers-in-law who forbid it because they fear something might happen*,* even though we’ve explained everything. But still*,* it’s difficult to change their minds.”* Health provider_06, endline, female midwife with diploma *“When my wife was offered to take IPTp-DP during her check-up at the healthcare facility*,* she was unsure and asked me. I advised her not to take it until there is proof or a recommendation from a doctor stating that it’s mandatory for pregnant women to take IPTp-DP.”* Husband_10, endline, 42, primary school education, 3 children

## Discussion

This is the first qualitative study to explore pregnant women’s acceptability of IPTp-DP in the context of a pilot programme in routine settings in Papua, Indonesia. The results demonstrate a positive shift in pregnant women’s attitudes towards IPTp-DP over time driven primarily by their desire to prevent malaria infection and reassurance that DP was safe to take presumptively to prevent malaria during pregnancy. These changes were catalysed by enhanced communication and messaging from healthcare providers, the resultant support from husbands and family members, and improved management of side effects at home. This study underscores the need for strong provider training and routine quality improvement to trouble shoot emergent barriers to uptake. Effective communication skills among providers proved crucial in improving women’s acceptability to IPTp-DP, a key factor in the successful introduction of the new strategy. These findings provide important insights for policy makers and programme managers seeking to optimise scale-up of IPTp-DP in similar settings.

Information and reassurance from healthcare providers contributed to women’s positive perceptions and helped ease their concerns regarding presumptive use of antimalarials for chemoprevention in pregnancy. Women’s reluctance to take IPTp-DP without clear information about the safety of repeated monthly courses of DP underscores the importance of effective communication about the intervention. Some providers in this study indicated the CQI training and monthly meetings gave them the tools to properly communicate with women about IPTp-DP and ultimately convince them to take it up. Evidence from varied settings suggests that quality improvement approaches can be effective when facility (in Indonesia) [18] or community-based providers (in South Africa) [19] are involved in identifying the issues and shaping the solutions. Interestingly, in this study, providers themselves described feeling more confident about the strategy following training, which provided them with reassurance about the safety and benefits of IPTp-DP. This is a key insight given earlier findings from the clinical trial in Indonesia where healthcare providers expressed strong reservations about giving DP presumptively without a diagnostic test [8]. Supportive interventions that enhance healthcare provider confidence in the safety of IPTp-DP and the benefits of the strategy, as well as how to communicate that information to women, will be essential for optimising national scale-up [10, 11].

For some women, side effects impeded uptake and continuation of monthly IPTp-DP courses. Information from healthcare providers about what side effects to expect and guidance on how to minimise them can mitigate this challenge, a finding consistent with studies of IPTp-DP conducted in Kenya [11] and with IPTp-SP in Ghana [20]. In addition to information, support from family members (particularly husbands) was an important dimension of acceptability. The role of male partner support for IPTp uptake has been documented in Nigeria [21] and Mozambique [22]. Home visits from providers also played a key role in reassuring women and their family members, giving them confidence to complete the doses. However, for this supportive strategy to be scalable additional human resources would be required. Interestingly, among women in the pilot evaluation who were given the correct number of DP tablets at ANC, adherence to the 2nd and 3rd doses taken at home was 90.3% [12], a finding that suggests side effects can be effectively managed at home with adequate support. Findings from our study indicate that a policy shift to IPTp-DP is acceptable to pregnant women provided they, and their family members, are well informed and adequately supported.

This study was conducted in the context of a pilot implementation reflecting acceptability under ‘real life’ conditions, including a national stock out of DP that temporarily halted the delivery of IPTp-DP during the first round of data collection. Drug stock outs are an established barrier to IPTp-SP uptake across sub-Saharan Africa [23], and a study in Ghana noted that stock outs can undermine trust and acceptability in the intervention [20]. Although women in this study did not report a loss of trust as a result of this disruption, health providers feared the stockout would damage trust in the programme and noted that stable supply of DP was essential for successful implementation of the new strategy.

### Strengths & limitations

This study was limited and enhanced by several factors. Social desirability bias could apply across the different participant groups as some individuals may have felt compelled to respond positively about the programme to please the interviewers. Although data was collected at two points (mid and endline), a baseline understanding of acceptability towards the intervention could have enhanced the findings. The study is strengthened by the inclusion of a range of participants from the health sector and community, enabling a diverse set of perceptions with which to explore acceptability.

## Conclusions

Women valued the opportunity to prevent malaria in pregnancy with IPTp-DP despite initial concerns about side effects and taking antimalarials presumptively during pregnancy, without a diagnostic test. Information from healthcare providers about the safety of DP and how to manage side effects were effective in addressing these concerns. Community engagement and sensitisation with husbands meant women were supported to take up the new strategy. Strengthening healthcare provider communication skills prior to national scale up is critical. These findings provide key insights for policy makers seeking to shift from a screen and treat strategy to preventive treatment for malaria in pregnancy.

## Supplementary Information

Additional File 1.

Additional File 2.

Additional File 3.

Additional File 4.

## Data Availability

Per our ethical approval constraints, individual participant qualitative data from this study will not be publicly available. The datasets generated and/or analysed during the current study can be made available from the corresponding author on reasonable request.

## Abbreviations

ANC: Antenatal care
CQI: Continuous quality improvement
DOT: Directly observed therapy
DP: Dihyroartemisninin–piperaquine
IDI: In–depth interview
IPTp: Intermittent preventive treatment in pregnancy
MoH: Ministry of Health
SP: Sulphadoxine–pyrimethamine
SST: Single screen and treat
WHO: World Health Organization
