# Supplementary material for: Pregnant women’s acceptability of intermittent preventive treatment with dihydroartemisinin-piperaquine from user and provider’s perspectives: qualitative findings from the pilot implementation in Papua, Indonesia
Source: BMC Pregnancy Childbirth. 2026 Apr 6;26:536. doi: 10.1186/s12884-026-09036-x (PMC13188372; doi:10.1186/s12884-026-09036-x)
Supplement: Supplementary file 2 — Additional File 2. [file 12884_2026_9036_MOESM2_ESM.docx]

## Midline topic guide: In-depth interview with health managers (Head of Facility, District or sub-district Health Management Team)

| Interview information |  |
| --- | --- |
| Informant ID : |  |
| Interviewer Name : |  |
| Health Facility or Institution : |  |
| Sub-district or district : |  |
| Date: |  |
| Start time: |  |
| End time: |  |
|  |  |
| Informan characteristic |  |
| Age : |  |
| Sex : |  |
| Highest professional qualification : |  |
| Tasks performed/role : |  |
| Length of time in current role (month) : |  |

| GUIDE | NOTES |
| --- | --- |
| 1. GENERAL INFORMATION   Currently   1. What is your role in the healthcare facility? 2. What are your duties (with that role) in the healthcare facility? 3. Where else do you work (practice) other than in this healthcare facility?   Before your current job  1. What was your previous job?  2. Have you ever worked in the ANC service department in another location? If so, where?  3. What were your involvements in those tasks? How long did you work there? |  |
| 1. WARM-UP   Main diseases in the area   1. What are the most common diseases in this area? 2. What are the main diseases that require special attention to be treated? 3. Where does malaria rank in the priority of disease management at the Puskesmas? |  |
| 1. PILOT IMPLEMENTATION OF IPTP-DP   What do you know about the IPTP-DP pilot implementation program from the Ministry of Health?   1. What do you know about the ongoing IPTP-DP pilot implementation program in ANC services? 2. Since you've been involved in the IPTP-DP, have you noticed any changes in healthcare services?   Probe: opinion on changes in IPTP-DP?   1. What is needed for the program to succeed? 2. In your opinion, where should IPTP-DP be implemented? Why? What is needed to expand the program? 3. Have you noticed any changes in pregnant women since the IPTP-DP implementation began? 4. Probe: behavior of pregnant women in ANC, reduction in malaria cases in pregnant women, coverage of IPTP-DP |  |
| Feasibility and expansion   1. The IPTP-DP program is still in the pilot implementation phase and will end around April 2023. What is your opinion if IPTP-DP is continued or if it reverts back to malaria screening at the first visit (K1) / SST when the pilot implementation ends? 2. Why do you think that?   Probe  ⮚ For pregnant women  ⮚ For healthcare facilities / health departments  ⮚ For malaria-endemic areas in Indonesia   1. What do you see as the potential negative impact of this change? Do you have any experiences to share?   Probe National  ⮚ Your area  ⮚ In healthcare facilities   1. What are the implications for:   ⮚ Financing  ⮚ Workforce, for example, training  ⮚ Information system  ⮚ Logistics  ⮚ Healthcare services |  |
| Additional questions, concerns, or comments Thank you for your participation in this study. Do you have any additional questions or comments to add? |  |

## Midline topic guide: In-depth interview with healthcare providers

| **Interview information** |  |
| --- | --- |
| Informant ID : |  |
| Interviewer Name : |  |
| Health Facility or Institution : |  |
| Sub-district or district : |  |
| Date: |  |
| Start time: |  |
| End time: |  |
|  |  |
| **Informan characteristic** |  |
| Age : |  |
| Sex : |  |
| Education : |  |
| Profession : |  |
| Position : |  |
| Length of work in this healthcare facility (months) : |  |
| Length of duty related to IPTP-DP (months): |  |

| **GUIDE** | **NOTES** |
| --- | --- |
| 1. GENERAL INFORMATION RELATED TO HEALTH WORKERS   Currently   1. What is your role in the healthcare facility? 2. What are your tasks (with that role) in the healthcare facility? 3. Where else do you work (practice) besides this healthcare facility?   Before current job   1. What was your previous job? 2. Have you ever worked in the ANC service department in another place? If so, where? 3. What were your involvements in those tasks? 4. How long did you work there? |  |
| **B. MALARIA**  **Main disease in the area**   1. What are the most common diseases in this area? 2. What is the main disease that requires special attention to be treated? 3. Where does malaria rank in the priority of disease management at the Puskesmas?   **Malaria Officer**  11. What is the condition of malaria in your Puskesmas? Based on the esismal report. Probe: Is there a trend of improvement or decline? Why?  **Pharmacy Officer**   1. What is the availability of malaria drugs currently? Probe: When will they be available again? 2. Are there any changes in the malaria treatment regimen?   Perception of malaria in pregnancy   1. What happens if a woman gets malaria while she is pregnant?   Probe   - - What happens to her baby?   - What is the best way to prevent malaria in pregnancy?   - What other ways are there to prevent malaria in pregnancy?  1. What do you think is more important - prevention or treatment? Why? 2. What do people in this area do when a pregnant woman is infected with malaria? |  |
| C. IPTP-DP: INTERVENTION   1. Please describe what the periodic malaria prevention program with anti-malaria drugs (IPTP-DP) is.   Probe   - What is done in IPTP-DP? - How does it work? - Do you have experience with other drugs for malaria prevention in pregnancy?  1. How is IPTP-DP administered? Probe  - Who is involved? - What needs to be prepared? - What is your opinion on administering IPTP-DP with direct supervision? What are your reasons? What are the challenges you face in monitoring mothers taking the medicine? - (Contraindications) Are all pregnant women given this drug? Probe: Are there certain conditions that make it impossible to administer IPTP-DP? Why? - (Exclusion) Are there any types of pregnant women who cannot participate? Why? - Have you ever encountered problems in administering IPTP-DP according to the guidelines? Can I know what problems you have encountered? - I want to emphasize again that this question will not affect performance evaluation. Because you have another role (according to the answers in general information), since the IPTP-DP program is new, do you feel that administering IPTP-DP is burdensome? What is your reason?   **PIC of Malaria**   1. Do you think the implementation of IPTP-DP in the Puskesmas is as expected? Are there any difficulties in the technical implementation of IPTP-DP? Probe: reporting and recording processes, drug supply 2. Are there any differences between treating malaria in pregnant women and other patients? Probe: related to drugs, DOT, home monitoring, CHW, etc.   **PIC of Pharmacy**   1. How is the implementation of IPTP-DP according to the IPTP-DP guidelines through the Pharmacy Management SOP (planning, storage, distribution, and recording)? 2. Do you think the implementation of IPTP-DP is in line with expectations or scientific knowledge? Probe: For example, administering DHP drugs for prevention by midwives in KIA rooms or Pustu and Posyandu; related to drug      1. Are there any special conditions or standards that must be met to allow other health workers to administer DHP drugs? Especially midwives.   **Information conveyed to pregnant women in IPTP-DP**   1. Malaria and Pharmacy PIC: What is your involvement in IPTP-DP socialization activities? 2. Can you describe how pregnant women are given information on IPTP-DP in your health facility   Probe on:   - Individual versus group information - When and where the information is given  1. In ANC services, there are many components that must be done. Regarding IPTP-DP, What job aids and/or guidance do you have to help you decide what a pregnant woman should be given?  - How does this help you? - Where do you keep them? - Where did you get these job aids and guidance from? - How accessible are these job aids and guidance? - Why were these job aids and guidance useful? Not useful? - What difference did these job aids and guidance make? - Do Pustu/Posyandu also have similar guidelines or tools? - Do you think that they should be given to other health facilities? Would this be useful to them? For what reason? - What would you suggest to make these job aids and guidance more useful to you?  1. What information are women given on IPTP-DP? (also ask to Pharmacy PIC)? Probe for information given on:  - Dose/duration - Number of courses/frequency - Date of return to ANC - Take with food or without food - Side effects - How to deal with side effects  1. How do you think pregnant women feel about IPTP-DP?   **Side-effects**   1. Have you seen women with side effects to IPTP-DP-DHP? What were these side effects?  - What did you do? - Do women usually have the same side effects with a second dose? - Do the side effects differ by gestation or number of months pregnant (gestational age)?  1. When should pregnant women be told about the possibility of experiencing side-effects to IPTP-DP?  - Have you experienced telling pregnant women about the side effects of IPTP-DP? What did you tell them? How did they react to the information? - Have you ever not given IPTP-DP because you were worried about side effects? Can you tell me about an example of this experience? What were the particular side effects you were worried about? And what was the reason that you were particularly worried with this woman in your example?   **Improving the quality of IPTP-DP services**   1. How do you feel about DHP being used for IPTP-DP in addition to treatment? 2. Is there anything that you can suggest that you feel will improve the giving of IPTP-DP? 3. Can you tell me about any training you have been given to help you in providing IPTP-DP?   Probe on   - Were they trained to give DHP for IPTP-DP? How and when were they trained?   **Adherence to completing doses at home**   1. Do you think that pregnant women complete their doses at home? 2. What influences whether pregnant women take the pills that they are given to take at home? 3. What influences whether women complete their IPTP-DP doses at home and why? 4. Do you actualy get to know whether pregnant women complete their doses? How do you get to know?   Probe: Strategies for monitoring drug intake at home, and their challenges. |  |
| **D. IPTP-DP PILOT PROGRAMME**  What do they know about the IPTP-DP pilot programme from the Ministry of Health?   1. What do you know about the ongoing IPTP-DP pilot program in ANC services? 2. Since you were involved in IPTP-DP activities, have you noticed any changes in health services?   Probes: opinions on changes in IPTP-DP?   1. What is needed for the program to be successful? 2. In your opinion, where should IPTP-DP be held? Why? What is required for the program to expand? 3. Have you noticed any changes in pregnant women since the IPTP-DP pilot started?   Probes: pregnant women's behavior in ANC, reduction of malaria cases in pregnant women, IPTP-DP coverage  **Feasibility and scale-up**   1. The IPTP-DP program is still in pilot implementation which will end around April 2023. What do you think if IPTP-DP is continued or returns to malaria screening at the first visit (K1) / SST when the pilot implementation ends? 2. What do you think about giving IPTP-DP at other health facilities in Indonesia?   Probe: in another district of Timika where malaria is endemic   1. What could be the challenges if implemented in other districts? 2. ​​What is needed for a successful expansion? 3. What needs to be changed for program expansion? 4. What impact might arise in daily activities if SST is replaced with IPTP-DP?   probes   - Positive change - Negative changes |  |
| **E. CONTEXT, MECHANISM AND OUTCOME**  Thank you for the answers and experiences that have been shared with me.  I’m going to read some general statements. After each statement I will pause, and I would like you to tell me what you think about the statement. These statements are not a reflection of what you do but they are statements to help us understand why IPTP-DP might be accepted or not accepted by health workers and pregnant women.  **Ask about whole sentences first and then about CONTEXT only; And then MECHANISM only**  **After each question ask**  Do you agree or disagree with this statement?  Why do you agree with it? / Why don’t you agree with the statement?  **Probe as needed:** Can you elaborate? Can you describe what you mean? Can you give me an example?  **Nature of the treatment (its purpose & components or activities comprising it)**   1. Health workers are used to giving the SST strategy (C) therefore they believe that a diagnostic test is essential to confirm pregnant women have parasites before giving them antimalarials (M)   Agree/Not agree; why?  **Dose**   1. Health workers do not usually give weight-based doses of drugs (C) therefore health workers find the DHP dosing complicated and confusing (M)   Agree/Not agree; why?  **Schedule**   1. Cinics are sometimes very busy (C) therefore health workers sometimes do not give women full information on how to take the drugs at home (M)   Agree/Not agree; why?   1. Women are sometimes very late or have a short time at the clinic (C) therefore health workers are sometimes unable to give them full information on how to take the drugs at home (M)   Agree/Not agree; why?  **Mode of delivery**   1. Health workers struggle to give the first IPTP-DP dose by DOT (C) therefore health workers prefer to give pregnant women all doses of IPTP-DP to take at home (M)   Agree/Not agree; why?    **Benefits (i.e., effectiveness in producing outcomes)**   1. DHP is known to be a good drug for treating malaria (C), therefore health workers feel that it is a good drug for IPTP-DP (M)   Agree/Not agree; why?   1. Pregnant women think that DHP is a very good drug for treating malaria (C) and therefore they save some of the DHP for when a family member has malaria (M) and do not take the full course of IPTP-DP (O).   Agree/Not agree; why?  **Risks (discomfort or side-effects experienced as a result of treatment)**   1. Health workers have experienced women complaining of side effects to IPTP-DP (C) therefore health workers believe that IPTP-DP causes side effects when women have not eaten (M) and do not give the first dose of IPTP-DP by DOT (O).   Agree/Not agree; why?   1. Health workers have experienced patients complaining of side effects to DHP (C) therefore some health workers might prefer to SST after confirming presence of malaria (M)   Agree/Not agree; why? |  |
| **Additional questions, concerns or comments**  Thank you very much for talking with me today, Do you have any questions or comments to add? |  |

## Midline topic guide: In-depth interview with pregnant women

| **Interview information** |  |
| --- | --- |
| Informant ID : |  |
| Interviewer Name : |  |
| Health Facility : |  |
| Date: |  |
| Start time: |  |
| End time: |  |
|  |  |
| **Informant characteristic** |  |
| Sub-district or district : |  |
| Age (year) : |  |
| Education : |  |
| Tribe : |  |
| Religion : |  |
| Gestational age (month) : |  |
| Sequence of pregnancies (including this pregnant): |  |
| Number of children born: |  |
| Marital status: |  |
| Number of ANC visits (this pregnancy) : |  |
| Number of IPTP-DP obtained : |  |
| Ever had malaria: |  |

| **GUIDE** | **NOTES** |
| --- | --- |
| 1. **ABOUT PREGNANCY** 2. Current condition and history of pregnancy 3. History of previous pregnancy. 4. Where have you checked your current pregnancy? How many times? 5. What is the importance of prenatal check-ups at health facilities? 6. What are you worried about during pregnancy? 7. If you were sick, what were the symptoms or conditions that prompted you to decide to visit a health facility? |  |
| 1. **MALARIA EXPERIENCE** 2. What do you know about malaria? 3. Have you or any of your family members/housemates ever had malaria? What was your or their experience when infected with malaria? Probe: Last experience on malaria treatment : Where did you seek treatment? What medication did you take? 4. What do you think about the condition of malaria in Timika or your area? 5. Can malaria be prevented? 6. How do you prevent malaria? What is your opinion about malaria prevention? |  |
| 1. **MALARIA AND MALARIA IN PREGNANCY** 2. What do you know about malaria during pregnancy?   Probe:   - Is malaria different when not pregnant? How can it be different?  1. Have you or any of your relatives ever had malaria during pregnancy? What did you or they experience? 2. Do pregnant women feel sick? And how does the sickness differ from malaria during pregnancy? 3. How do you prevent malaria during pregnancy? What is your opinion on preventing malaria during pregnancy during ANC (antenatal care)?   D. MATERNAL HEALTH SERVICES   1. What was the reason for your first visit to ANC during this pregnancy/your previous pregnancy?   Probe:   - Was the visit for routine check-up or because you were feeling sick?  1. What kind of examinations and services did you receive? 2. Were you provided with everything you needed? Is there anything else you wanted? Probe:  - Would you prefer to go for check-ups somewhere else? By whom? Why? |  |
| 1. DISCUSSION ON ANC VISITS RELATED TO MALARIA PREVENTION   I would like to ask you what you remember about your first ANC visit (possibly receiving RDT or IPTP-DP) during your previous pregnancy.   1. (If you have been pregnant before) In your previous pregnancy, were you ever screened for asymptomatic malaria during ANC at the health center (before IPTP-DP was introduced)? Please share your experience with previous ANC visits. 2. In your current pregnancy, have you also been screened for asymptomatic malaria during your first ANC visit? 3. Have you ever been given IPTP-DP for malaria prevention? Probe on differences in services and perceptions of these services.     I would like to ask some questions about the medications given during your first ANC visit in this pregnancy.   1. What medication was given? Do you know the names? What color are the medications? Probe: For each medication, ask about the number of tablets and the size of the tablet. 2. What are the medications for? 3. When and where did you take the medications? 4. Probe about the blue medication for malaria prevention - How do you feel about the malaria prevention medication? Probe: What do you like about it? What do you not like about it? 5. How do you feel when taking the medication? 6. What complaints did you have after taking the medication? What did you do about the complaints? |  |
| 1. **FOLLOW-UP ANC VISITS**   [*To find out what they remember about what happened on return visits to ANC*]   1. When was your last prenatal check-up? Where did you go? (or recall from earlier questions). 2. Tell me about the services you received during the follow-up visit. Probe: Were these services what you needed? Did you have any unanswered concerns or expectations about specific medications or examinations? 3. What was different about the follow-up visit compared to the first visit during this pregnancy? 4. What did you like about the follow-up visit? Why? 5. What did you not like about what happened during the follow-up visit? Why? 6. After the follow-up visit, do you feel the need to schedule another visit? Why? 7. (if previously pregnant) During your previous pregnancy, did you visit the health center more than once? If so, how does the follow-up visit during this pregnancy compare to the previous one? |  |
| G. IPTP-DP   1. Have you ever heard of IPTP-DP during ANC? What do you know about it? 2. What is your opinion about anti-malaria medication/blue medication for malaria prevention during pregnancy? Probe  - How does it work? / Why is it effective? - Is IPTP-DP beneficial for the mother and the baby? Why?  1. What was your experience when receiving IPTP-DP? Probe  - What medication did you receive? By whom? Where? - How many tablets did you take per day and for how many days? DescrIPTP-DPion of the tablets given - color, size, etc. - How did you take the first tablet? Probe - Directly Observed Treatment (DOT) at health facilities - What is your opinion about taking medication under the supervision of healthcare workers? - How did you feel when taking IPTP-DP (good and/or bad) (further probing if afraid of harming the baby)? - Would you take IPTP-DP again in the future? Why? - (based on previous answer regarding SST) Feelings about taking IPTP-DP compared to malaria screening (SST).   Compliance to complete the dose at home   1. Is the medication (IPTP-DP) taken again the following day? Probe: How is the medication given? Is it taken home or delivered by healthcare workers? 2. If delivered by healthcare workers, how did you feel? 3. Did you take all of the medication as directed? Why?  - Note: further probing if refusal is due to spouse or family member's objection, and what she thinks to solve the problem. - Do you still have the remaining medication? What are your plans for the medication?  1. If you have received IPTP-DP more than once, how do you feel compared to your previous experience of taking IPTP-DP? Probe: related to concerns.   Side effects   1. How was your experience after taking IPTP-DP medication? Probe:  - What did you experience? - Did you experience the same thing as when you took IPTP-DP before?  1. What was explained by healthcare workers, if any, regarding possible side effects after taking the medication? 2. Did healthcare workers explain what to do if you experience any side effects (no. 38)? What did they suggest? |  |
| **H. CONTEXT, MECHANISM, AND OUTCOME**  INTRODUCTION  Thank you for sharing your experience. After this, there are several statements that I am going to read and I would like to hear your opinion.  Regarding the prevention of malaria in pregnancy, not all pregnant women comply with taking all the medication given for malaria prevention in pregnancy.  Do you agree or disagree? And why? Do you have any experience that you can share about this?  (Your answer to this question will not be judged)   1. The number of tablets that must be taken to prevent malaria in pregnancy is too many, therefore not all tablets are taken. Agree/Disagree; why? 2. Medication to prevent malaria in pregnancy must be taken repeatedly (every 4 weeks) and therefore not all tablets are taken. Agree/Disagree; why? Probe:  - How often and how many tablets do you prefer to take? - Probe: every day, or every month  1. The tablet size for preventing malaria in pregnancy is too large and therefore not all tablets are taken. Agree/Disagree; why? 2. Medication to prevent malaria in pregnancy makes pregnant women feel sick and therefore not all medication is taken. Agree/Disagree; why? 3. Pregnant women like to share medication and therefore do not take all the medication to prevent malaria in pregnancy. Agree/Disagree; why? 4. Pregnant women sometimes forget to take medication to prevent malaria in pregnancy and therefore not all medication is taken. Agree/Disagree; why? 5. Medication to prevent malaria in pregnancy is very good for treating malaria and therefore some pregnant women keep the medication for family members who have malaria and therefore not all medication is taken. Agree/Disagree; why? 6. According to you, what are other reasons why some pregnant women do not take all the medication given to prevent malaria during pregnancy? 7. In your opinion, what can be done to ensure that pregnant women take all the medication given to prevent malaria during pregnancy? |  |
| **Additional questions, concerns, or comments**  Thank you very much for talking with me today. Do you have any additional questions or comments to add? |  |

## Endline topic guide: In-depth interview with health managers (Head of Facility, District, Province, and National Health Management Team)

| **Interview information** |  |
| --- | --- |
| Informant ID : |  |
| Interviewer Name : |  |
| Health Facility or Institution : |  |
| Sub-district or district : |  |
| Date: |  |
| Start time: |  |
| End time: |  |
|  |  |
| **Informant characteristics** |  |
| Age : |  |
| Sex : |  |
| Highest professional qualification : |  |
| Tasks performed/role : |  |
| Length of time in current role (month) : |  |

| **GUIDE** | **NOTES** |
| --- | --- |
| 1. **GENERAL INFORMATION**   Currently   1. What is your role in the healthcare facility? 2. What are your duties (with that role) in the healthcare facility? 3. Where else do you work (practice) other than in this healthcare facility?   Before your current job   1. What was your previous job? 2. Have you ever worked in the ANC service department in another location? If so, where? 3. What were your involvements in those tasks? How long did you work there? |  |
| 1. **WARM-UP** 2. What are the most common diseases in this area? 3. Where does malaria rank in the priority of disease management at the Puskesmas?    1. If malaria is considered high burden in the community – probe why? (number of cases, poverty, lack of prevention knowledge, other) |  |
| 1. **PILOT IMPLEMENTATION OF IPTP-DP** 2. What do you know about the ongoing IPTP-DP pilot implementation program in ANC services? 3. Since you have been involved in IPTP-DP activities, have you noticed any changes in ANC service delivery or utilisation?    - Changes in pregnant women's behavior towards ANC? (ie. attendance)    - Changes in IPTp coverage? 4. Have you noticed any benefit to pregnant women’s health since the IPTP-DP pilot started?   Probes: reduction of malaria cases, improved birth outcomes   1. What is needed for the IPTp-DP program to be successful? Probe: What other measures are being taken to increase awareness and acceptance of IPTp-DP among the community, especially pregnant women? For example, health promotion activities, ensuring the availability of medication supplies, providing training support, enhancing staff coordination (leadership), etc. 2. In your opinion, where should IPTP-DP be implemented? Why? What is needed to expand the program in this district/sub-district [name of district/sub-district]? |  |
| **Feasibility and expansion**  The IPTP-DP program is still in the pilot implementation phase and will end around September 2023.   1. Do you think IPTP-DP should be continued when the pilot implementation ends?   Probe: IPTp alone or with malaria screening at the first visit (K1) / SST? Or revert to SST alone?   - If you still want SST, is it only 1^st^ trimester or at every ANC visit etc.(IST) - Why? (the preferences)  1. Why do you think that (response to Q14)?   Probe reasons that involve: For pregnant women, For healthcare facilities / health departments, For malaria-endemic areas in Indonesia   1. Do you envisage any challenges of expanding IPTp? Do you have any experiences to share?   Probe: National, your area, in healthcare facilities   1. What are the implications for:  - Financing (e.g., cost of training, procurement etc., cost-benefits/effectiveness [savings made on cases prevented, reductions in LBW wtc.] - Governance (e.g., expansion to village midwives or community health workers) - Health Workforce, (e.g., pre-service and in-service training, staff attrition and therefore knowledge attrition, supervision, success or otherwise of CQI, QA etc. - Information system (e.g., granularity of data by puskesmas, pustu and posyendu levels, drug quantification) - Medical products/drugs including supply (e.g. drug resistance due to DP used for treatment and prevention) - Healthcare service delivery (mode of delivery – thoughts on all the variations observed in the pilot). Where? Whom? |  |
| **Additional questions, concerns, or comments** Thank you for your participation in this study. Do you have any additional questions or comments to add? |  |

## Endline topic guide: In-depth interview with health providers

| **Interview information** |  |
| --- | --- |
| Informant ID : |  |
| Interviewer Name : |  |
| Health Facility or Institution: |  |
| Sub-district or district : |  |
| Date: |  |
| Start time: |  |
| End time: |  |
|  |  |
| **Informant characteristics** |  |
| Age : |  |
| Sex : |  |
| Education : |  |
| Profession : |  |
| Position in facility: |  |
| Length of work in this healthcare facility (months) : |  |
| Length of duty related to IPTP-DP (months): |  |

| **GUIDE** | **NOTES** |
| --- | --- |
| 1. **GENERAL INFORMATION RELATED TO HEALTH WORKERS**   Currently   1. What are your tasks (with that role) in the healthcare facility? 2. Where else do you work (practice) besides this healthcare facility? |  |
| 1. **MALARIA**   **Main diseases in the area**   1. What are the most common diseases in this area? 2. Where does malaria rank in the priority of disease management at the Puskesmas?    1. If malaria is considered high burden in the community – probe why? (number of cases, poverty, lack of prevention knowledge, other)   **Malaria Officer**   1. What is the condition of malaria in your Puskesmas? Based on the esismal report. Probe: Is there a trend of improvement or decline? Why?   **Pharmacy Officer**   1. What is the availability of malaria drugs currently? For treatment? For prevention? Probe: When will they be available again?   **Perception of malaria in pregnancy**   1. What happens if a woman gets malaria while she is pregnant?   Probe   - - What happens to her baby?   - What is the best way to prevent malaria in pregnancy?   - What other ways are there to prevent malaria in pregnancy?  1. What do you think is more important - prevention or treatment? Why? 2. What do people in this area do when a pregnant woman is infected with malaria? |  |
| **C. IPTP-DP: INTERVENTION**   1. Please tell what you know about the use of anti-malaria drugs for prevention in pregnancy (IPTP-DP).  - When it should be given (trimester, timing interval, number of times etc.)? - What happens if a woman in second trimester has not yet had SST? (probe: do you still screen women for malaria even if they will receive IPTp? Ie. women in the 2^nd^ trimester) - What are the benefits of IPTp-DP? - Do you have experience with other drugs for malaria prevention in pregnancy?  1. How is IPTP-DP administered?  - Who delivers IPTp to the women at ANC? - How do you administer IPTp-DP? Walk me through each step in the administration of IPTp to a pregnant women. - What is your opinion on administering IPTP-DP to women under observation (DOT)? What are your reasons? What are the challenges you face in monitoring whether mothers take the medicine? - (Contraindications) Are all pregnant women given this drug? Probe: Are there certain conditions that make it impossible to administer IPTP-DP? Why? - (Exclusion) Are there some pregnant women who are not able to access IPTp DP? If so, can you explain why? (probe issues related to access/equity, and socio-cultural factors) - Have you ever encountered any issues in administering IPTP-DP according to the guidelines? Can you explain the problems you have encountered? - I want to emphasize again that this question will have no bearing on your job. Because you have another role (according to the answers in general information), since the IPTP-DP program is new, have you had any challenges in administering IPTP-DP? Please describe these challenges and how you overcame them?   **Information conveyed to pregnant women in IPTP-DP**   1. **Malaria and Pharmacy PIC**: What is your involvement in creating awareness among pregnant women on IPTP-DP as part of socialization activities? Probe (when, and how)   **Midwife**   1. Can you describe how pregnant women are **given information on IPTP-DP** in your health facility  - Individual versus group information - When and where the information is given  1. What job aids and/or guidance do you have to help you decide **when to administer IPTp DP**?  - What information do they have that is most useful for you? - Where are the job aids kept at your facility? Are they accessible? - Where did you get these job aids and guidance from? - Why were these job aids and guidance useful? Not useful? - Do Pustu/Posyandu also have similar guidelines or tools? - Do you think that they should be given to other health facilities? Would this be useful to them? For what reason? - What would you suggest to make these job aids and guidance more useful to you?  1. What information do you give women before giving them IPTP-DP? (also ask to Pharmacy PIC)? Probe for information given on:  - Dose/duration - Number of courses/frequency - Date of return to ANC - Take with food or without food - Side effects - How to deal with side effects  1. How do you think pregnant women feel about IPTP-DP?    1. Do you have pregnant women who took IPTp DP once but then refused to take it again? Why did they not want to take it again?    2. What would help encourage pregnant women to take IPTp DP again? 2. What do you think would improve pregnant women’s acceptability of IPTp DP? Please expand. 3. Do you think pregnant women would be/are supported by family/friends to take IPTp-DP? Why or why not?    1. What about support from husbands?   **Side-effects**   1. Have you seen women with side effects to IPTP-DP? Or have women reported side effects to you during their next ANC visit? Can you explain?    1. What were these side effects?  - What did you do/what did you advise them?  1. Do women usually have the same side effects when they take IPTp DP again at the next ANC appointment?    - Why do you think they experience the same or different side effects the next time they have IPTp DP?  - Do the side effects differ by gestation or number of months pregnant (gestational age)?  1. When women come for their next ANC **or** IPTp DP dose, do they talk to you about the side effects they experienced with the previous IPTp DP? What do they say?    1. Do they want to take the next dose? What do you think are their reasons for this? 2. When should pregnant women be told about the possibility of experiencing side-effects to IPTP-DP?  - Have you experienced telling pregnant women about the side effects of IPTP-DP? What did you tell them? How did they react to the information? - What do you tell women about how to manage side effects at home? - Have you ever not given IPTP-DP because you were worried about side effects? Can you tell me about an example of this experience? What were the particular side effects you were worried about? And what was the reason that you were particularly worried with this woman in your example?   **Adherence to completing IPTp-DP doses at home**   1. Do you think that pregnant women complete their DP doses at home? 2. What factors influence whether pregnant women finish both DP doses that they are given to take at home? 3. What do you think could help pregnant women finish the doses of DP at home? Why? 4. Is there a way to confirm if pregnant women complete their DP doses at home? How does that work?    1. Is there follow up to check they have finished their doses? What does that look like?    2. Can you describe some challenges in monitoring adherence to the doses taken at home? |  |
| **D. IPTP-DP PILOT PROGRAMME**  Now I will ask some questions about the implementation of IPTp-DP pilot programme by the Ministry of Health.   1. Since you have been involved in IPTP-DP activities, have you noticed any changes in ANC service delivery or utilisation?    1. Changes in pregnant women's behavior towards ANC? (ie. attendance)    2. Changes in IPTp coverage? 2. Have you noticed any benefit to pregnant women’s health since the IPTP-DP pilot started?    1. Probes: reduction of malaria cases, improved birth outcomes 3. In your opinion, where should IPTP-DP be delivered? Why? 4. What do you think is needed for this program to be successful? 5. Did your facility experience stock outs of DP during the pilot implementation of IPTp-DP?    1. If yes, what was the impact of the stockout on the IPTp programme?   **Improving the quality of IPTP-DP services**   1. How do you feel about DP being used for IPTP-DP (ie. for prevention) in addition to treatment? 2. Is there anything that you can suggest that you feel will improve the following:    1. Administration IPTP-DP?    2. Women’s adherence to DP?    3. Acceptability towards IPTp DP 3. Can you tell me about any training you have been given to help you administer IPTP-DP?  - How and when were you trained? - If they were trained, did you feel the training you received was adequate? Do you still have any knowledge gaps related to IPTp-DP? If so, what are they? - What are the implications of CQI?   **PIC of Malaria**   1. Do you think the implementation of IPTP-DP in the Puskesmas has been successful? Are there any difficulties in the implementation of IPTP-DP according to guidelines?    - With respect to reporting and recording processes (STT vs IPTp)    - With respect to drug supply (treatment vs prevention) 2. Are there any differences of services between treatment and prevent malaria in pregnant women? Probe: related to diagnosis, drugs, DOT, home monitoring, CHW, health information system, etc.   **PIC of Pharmacy**   1. How is the implementation of IPTP-DP according to the IPTP-DP guidelines monitored through the Pharmacy Management SOP (planning, storage, distribution, and recording)? 2. Where & by whom do you think the administration of IPTP-DP is appropriate?    - What about administering DP drugs for prevention by midwives in MCH rooms or Pustu and Posyandu? Can you explain your answer?    - Are there conditions or standards that must be to met to allow other health worker cadres such as village midwives and community health workers to administer DP to pregnant women?   **Feasibility and scale-up**  The IPTP-DP program is still in pilot implementation which will end around October 2023.   1. Do you think IPTP-DP should be continued when the pilot implementation ends?  - IPTp alone or with malaria screening at the first visit (K1) / SST? Or revert to SST alone? - if they still want SST, is it only in 1^st^ trimester or at every ANC visit?  1. What do you think about giving IPTP-DP at other health facilities in Indonesia?  - in another district of Timika where malaria is endemic?  1. What could be the challenges if implemented in other districts or regions? 2. What is needed for a successful expansion to improve access/equity/uptake among women? 3. What needs to be improved or changed for program expansion? 4. If SST is replaced with IPTP-DP, what changes might occur in the delivery of services at ANC (including at pharmacy)   probes   - Can you identify potential positive changes? - Can you identify potential negative changes? |  |
| **E. CONTEXT, MECHANISM AND OUTCOME**  Thank you for the answers and experiences that have been shared with me.  I’m going to read some general statements. After each statement I will pause, and I would like you to tell me what you think about the statement. These statements are not a reflection of what you do but they are statements to help us understand why IPTP-DP might be accepted or not accepted by health workers and pregnant women.  **Ask about whole sentences first and then about CONTEXT only; And then MECHANISM only**  **After each question ask**  Do you agree or disagree with this statement?  Why do you agree with it? / Why don’t you agree with the statement?  **Probe as needed:** Can you elaborate? Can you describe what you mean? Can you give me an example?  **Nature of the treatment (its purpose & components or activities comprising it)**   1. Health workers are used to giving the SST strategy (C) therefore they believe that a diagnostic test is essential to confirm pregnant women have parasites before giving them antimalarials (M). Agree/Not agree; why?   **Dose**   1. Health workers do not usually give weight-based doses of drugs (C) therefore health workers find the DHP dosage insufficient or too low (M). Agree/Not agree; why?   **Schedule**   1. Clinics are sometimes very busy (C) therefore health workers sometimes do not give women full information on how to take the drugs at home (M). Agree/Not agree; why? 2. Women are sometimes very late or have a short time at the clinic (C) therefore health workers are sometimes unable to give them full information on how to take the drugs at home (M). Agree/Not agree; why?   **Mode of delivery**   1. Health workers struggle to give the first IPTP-DP dose by DOT (C) therefore health workers prefer to give pregnant women all doses of IPTP-DP to take at home (M). Agree/Not agree; why?   **Benefits (i.e., effectiveness in producing outcomes)**   1. DHP is known to be a good drug for treating malaria (C), therefore health workers feel that it is a good drug for IPTP-DP (M). Agree/Not agree; why? 2. Pregnant women think that DHP is a very good drug for treating malaria (C) and therefore they save some of the DHP for when a family member has malaria (M) and do not take the full course of IPTP-DP (O). Agree/Not agree; why?   **Risks (discomfort or side-effects experienced as a result of treatment)**   1. Health workers have experienced women complaining of side effects to IPTP-DP (C) therefore health workers believe that IPTP-DP causes side effects when women have not eaten (M) and do not give the first dose of IPTP-DP by DOT (O). Agree/Not agree; why? 2. Health workers have experienced patients complaining of side effects to DP (C) therefore some health workers might prefer to give DP with SST, after confirming presence of malaria (M). Agree/Not agree; why? |  |
| **Additional questions, concerns or comments**  Thank you very much for talking with me today, Do you have any questions or comments to add? |  |

## Endline topic guide: In-depth interview with pregnant women

| **Interview information** |  |
| --- | --- |
| Informant ID : |  |
| Interviewer Name : |  |
| Health Facility : |  |
| Date: |  |
| Start time: |  |
| End time: |  |
|  |  |
| **Informant characteristic** |  |
| Sub-district or district : |  |
| Age (year) : |  |
| Education : |  |
| Tribe : |  |
| Religion : |  |
| Gestational age (month) : |  |
| Sequence of pregnancies (including this pregnant): |  |
| Number of children born: |  |
| Marital status: |  |
| Number of ANC visits (this pregnancy) : |  |
| Number of IPTP-DP obtained : |  |
| Ever had malaria: |  |

| **GUIDE** | **NOTES** |
| --- | --- |
| 1. **ABOUT PREGNANCY** 2. Current condition and history of pregnancy 3. Where have you attended ANC to have your current pregnancy checked? How many times? 4. What is the importance of prenatal check-ups at health facilities? 5. What concerns, if any, do you have during pregnancy? 6. If you experienced an illness/ sickness in your current pregnancy and sought treatment, what were the symptoms or conditions that prompted you to visit a health facility? |  |
| 1. **MALARIA EXPERIENCE** 2. What do you know about malaria? 3. Have you or any of your family members/housemates ever had malaria? What was your or their experience when infected with malaria? Probe: Last experience on malaria treatment : Where did you seek treatment? What medication did you take? 4. What do you think about the condition of malaria in Timika or your area? 5. Can malaria be prevented? 6. How do you prevent malaria? What is your opinion about malaria prevention? |  |
| 1. **MALARIA AND MALARIA IN PREGNANCY** 2. Have you or any of your relatives ever had malaria during pregnancy? What did you or they experience? 3. How do you prevent malaria during pregnancy? What is your opinion on preventing malaria during pregnancy during ANC (antenatal care)? 4. **MATERNAL HEALTH SERVICES** 5. What was the reason for your first visit to ANC during this pregnancy/your previous pregnancy?   Probe:   - Was the visit for routine check-up or because you were feeling sick?  1. What kind of examinations and services did you receive? 2. Were you provided with everything you needed? Is there anything else you wanted? Probe:  - Would you prefer to go for check-ups somewhere else? By whom? Why? |  |
| 1. **DISCUSSION ON ANC VISITS RELATED TO MALARIA PREVENTION & IPTp DP**   I would like to ask you what you remember about your first ANC visit (possibly receiving malaria test or IPTP-DP) during your current pregnancy and previous pregnancy (if applicable).   1. In your ***current pregnancy***, do you think have you been screened for malaria (SST/prevention) (meaning when you did not have symptoms of malaria) during your first ANC visit? Please share your experience about when you got the test (if more than once), how this test was done. 2. Have you ever heard of IPTP-DP during ANC? What do you know about it?  - In your current pregnancy, have you ever been given medication to prevent malaria?  1. What was your experience when receiving IPTP-DP? Probe:  - What drug did you receive? Can you describe the colour, size? - How many tablets did you take per day and for how many days? - Who gave you the medication? Where were you given the medication? (at home or clinic)? Probe: were you given all tablets at the health facility or were some tablets given to you at home – please describe. - How did you take the first tablet? Probe: Directly Observed Treatment (DOT) at health facilities - What is your opinion about taking medication under the observation of healthcare workers?  1. What is your opinion about anti-malaria medication/blue medication for malaria prevention during pregnancy? Do you think it is important to take? Why do you think this?  - How did you feel when taking IPTP-DP (good and/or bad) (further probing if afraid of harming the baby)? - What complaints did you have after taking the blue medication for malaria prevention? What did you do about the complaints? - Would you take IPTP-DP again in the future? Why do you say this? (**if they refused IPTp-DP when offered again at a subsequent ANC visit – please ask ADDITIONAL QUESTIONS**:   - Why did you refuse to take IPTp again?   - What would help make you willing to take IPTp again if you were offered it? - If DP/blue medication was not available at the health facility, would you be willing to purchase it from a pharmacy? Why or why not?   **Side effects**   1. How was your experience after taking IPTP-DP medication? Probe: did you have any side-effects:  - What did you experience? - How did you deal with the side effects?  1. Did you report the side effects to a health provider and if so, how did you do this? 2. Did healthcare workers explain what to do if you experience any side effects? What did they tell you? 3. What was explained by healthcare workers, if any, regarding possible side effects after taking the medication? 4. If you have received IPTp-DP more than once, how do you feel compared to your previous experience of taking IPTP-DP? Probe: related to concerns. 5. ADDIONAL QUESTION FOR PW who have received IPTp DP multiple times:    1. Did you experience the same side effects each time you took IPTp DP (for example: if you received IPTp DP at 3 separate ANC visits were your side effects the same each time or different)? If they were different, how?    2. Please explain why you think the side effects were different on the different occasions that you took IPTp DP. |  |
| **Compliance to completing the doses at home:**   1. Is the IPTP-DP taken again the following day? Probe (for each dose over 3 days): How is the medication given? Is it taken home from the facility or delivered at their home by healthcare workers? 2. If delivered by healthcare workers at home, how did you feel about being visited at home? 3. Did you take all of the DP for IPTp as directed? Why?  - Probe: is the refusal due to spouse or family member's objection? and what she thinks could help resolve this challenge.  1. How did the health workers know you took the doses at home?    1. Probe: did you receive any phone calls or home visits? 2. Do you still have the remaining DP? What are your plans for the DP? 3. What would help you to finish the full DP dose? Why? (probe reminders, advice to manage side effects, taking meds at night or with food, etc.)   **Strategy**   1. How do you feel about taking medication to prevent malaria in pregnancy?    1. Probe: would you prefer being *tested at the first visit and receiving medication only if you are positive* OR *taking medication to prevent malaria at each visit*?    2. Can you explain your answer? 2. You told us that you received each dose of IPTp by [repeat what they told you in Q26]. What do you think about this delivery strategy? (home visits by health provider vs phone calls vs self-administration). How could it be improved? |  |
| 1. **CONTEXT, MECHANISM, AND OUTCOME**   INTRODUCTION  Thank you for sharing your experience. After this, there are several statements that I am going to read and I would like to hear your opinion.  Regarding the prevention of malaria in pregnancy, not all pregnant women comply with taking all the medication given for malaria prevention in pregnancy.  Do you agree or disagree? Please tell us why you agree or do not agree? Do you have any experience that you can share about this?  (Your answer to this question will not be judged)   1. The number of tablets that must be taken to prevent malaria in pregnancy is too many, therefore not all tablets are taken. Agree/Disagree; why? 2. Medication to prevent malaria in pregnancy must be taken repeatedly (every 4 weeks) and therefore not all tablets are taken. Agree/Disagree; why? Probe:  - How often and how many tablets do you prefer to take? - Probe: every day, or every month  1. The tablet size for preventing malaria in pregnancy is too large and therefore not all tablets are taken. Agree/Disagree; why? 2. Medication to prevent malaria in pregnancy makes pregnant women feel sick and therefore not all medication is taken. Agree/Disagree; why? 3. Pregnant women like to share medication and therefore do not take all the medication to prevent malaria in pregnancy. Agree/Disagree; why? 4. Pregnant women sometimes forget to take medication to prevent malaria in pregnancy and therefore not all medication is taken. Agree/Disagree; why? 5. Medication to prevent malaria in pregnancy is very good for treating malaria and therefore some pregnant women keep the medication for family members who have malaria and therefore not all medication is taken. Agree/Disagree; why? 6. According to you, what are other reasons why some pregnant women do not take all the medication given to prevent malaria during pregnancy? 7. In your opinion, what can be done to ensure that pregnant women take all the medication given to prevent malaria during pregnancy? |  |
| **Additional questions, concerns, or comments**  Thank you very much for talking with me today. Do you have any additional questions or comments to add? |  |

## Endline topic guide: In-Depth Interview with Husbands of Pregnant Women

| **Interview Information:** |  |
| --- | --- |
| Informant ID |  |
| Interviewer Name: |  |
| Health Facility: |  |
| Date: |  |
| Interview Start Time:: |  |
| Interview End Time: |  |
|  |  |
| **Informant Characteristics:** |  |
| Sub-district or district: |  |
| Age (years)): |  |
| Education: |  |
| Primary Occupation: |  |
| Ethnicity: |  |
| Religion: |  |
| Number of live-born children: |  |
| Marital status: |  |
| History of malaria: |  |

| **GUIDES** | **NOTES** |
| --- | --- |
| **PREGNANCY**   - Current Pregnancy and Pregnancy History - How important do you think antenatal check-ups at health facilities are? Do you have any particular concerns? - Has your wife ever experienced illness or complaints during this pregnancy and sought medical treatment? |  |
| **MALARIA EXPERIENCE**   - What do you know about malaria? - Have you or your family/household members ever had malaria? What was your experience or theirs during malaria infection?   - Ask: Last experience receiving malaria treatment, where it was obtained, which medications were used - How would you describe the malaria situation in Timika or your area? - Can malaria be prevented? - How can malaria be prevented? What are your thoughts on malaria prevention? |  |
| **MALARIA AND MALARIA DURING PREGNANCY**   - Has your wife or any relatives ever had malaria during pregnancy? What was the experience like? What were your concerns? (Before PEMILA-OAM) - How can malaria be prevented during pregnancy? What is your opinion on preventing malaria during pregnancy within antenatal care? |  |
| **DISCUSSIONS ON ANC VISITS RELATED TO MALARIA PREVENTION AND IPTp-DP**   - Have you heard about PEMILA-OAM? What do you know about it? - In this pregnancy, has your wife been given medication to prevent malaria? - What do you think about your wife taking antimalarial medication/“blue medicine” for malaria prevention during pregnancy? Do you believe it is important? Why? - Do you have any concerns about your wife taking IPTp-DP (whether good or bad)? (Probe further if worried about harm to the baby.) - Would you allow your wife to take IPTp-DP again in the future? Why? (If they refused IPTp-DP again during antenatal visits.) - What would help convince you that your wife should take IPTp again if offered? - If DP/“blue medicine” was unavailable at the health facility, would you be willing to purchase it from a pharmacy? Why or why not?   **SIDE EFFECTS**   - When your wife returned home after taking IPTp-DP (malaria preventive medicine), did she complain of any issues? What did you do about it? - If your wife has taken IPTp-DP more than once, how was her experience compared to the previous times? - If your wife has received IPTp-DP multiple times: - Did she experience the same side effects each time? (For example, if she took it three times, were the side effects the same on each visit?) - If different, what were they and why? |  |
| **ADHERENCE TO COMPLETING THE DOSE AT HOME**   - Did your wife continue taking IPTp-DP the following day?   - Ask about each dose over the three days: how did she receive the medicine? Did she bring it home from the health facility or was it delivered to your home by health workers? - If it was delivered to your home, how did you feel about the home visit? - Does your wife have any leftover DP medicine? What does she plan to do with it? - What do you think could help your wife complete the full DP dose? Why?   Ask: reminders, advice on handling side effects, taking medication at night or with food, etc.  **STRATEGY**   - Would you prefer your wife to receive the medicine after being diagnosed with malaria or to take the medicine directly as a preventive measure at each antenatal visit? - What are your thoughts on the IPTp-DP implementation strategy? (Home visits by health workers vs phone calls vs self-administration) - How could these services be improved at health facilities? |  |
| **Additional Questions and Comments**   - Thank you very much for sharing with me today. Do you have any questions or comments? |  |
