# Supplementary material for: Pregnant women’s acceptability of intermittent preventive treatment with dihydroartemisinin-piperaquine from user and provider’s perspectives: qualitative findings from the pilot implementation in Papua, Indonesia
Source: BMC Pregnancy Childbirth. 2026 Apr 6;26:536. doi: 10.1186/s12884-026-09036-x (PMC13188372; doi:10.1186/s12884-026-09036-x)
Supplement: Supplementary file 3 — Additional File 3. [file 12884_2026_9036_MOESM3_ESM.docx]

**Coding Framework – pregnant women**

| **Theme** | **Sub-theme** | **Definition** |
| --- | --- | --- |
| Access | Availability | confidence and satisfaction with getting the services / medical supplies |
|  | Accessibility | convenient and difficult to go to health facilities or visited |
|  | Accommodation | satisfaction of waiting time and get in touch with the services |
|  | Affordability | satisfaction of services price, including health insurance |
|  | Acceptability | satisfaction of appearance and neighborhood |
| Acceptability | Affective attitude | how an individual feels about the intervention; **attitude** toward interventions |
|  | Burden | the perceived amount of **effort** that is required to participate in the intervention; reasons for discontinuation/ dropout |
|  | Ethicality / ethical consequences | the extent to which the intervention has good fit with an individual’s value system, associated with side effects |
|  | Intervention coherence | the extent to which the participant **understands** the intervention and how it works |
|  | Opportunity costs | the extent to which benefits, profits or values must be given up to engage in the intervention; influence on adherence and participation |
|  | Perceived effectiveness | the extent to which the intervention is perceived as likely to achieve its purpose |
|  | Self-efficacy | the participants’ confidence that they can perform the behaviour r(s) require to participate in the intervention; willingness to participate in the intervention |
| Context | Pregnancy experience | Condition or complaints during pregnancy |
|  | Malaria experience | Malaria experience, including malaria in pregnancy |
|  | ANC experiences | Type of services  Quality of care – satisfaction  Attitude of providers |
|  | Malaria prevention knowledge & use | IPTp-DP, SST, Bed nets |
| Adherence | IPTp experience | Woman’s description of her IPTp experience   - DOT + 2 doses at home - Not DOT and all 3 doses at home - No DOT + 3 doses home + home visit - No IPTp - DOT 1+DOT+2+DOT3 |
|  | Full adherence | Took all dose |
|  | Non-adherence | Did not take 2^nd^ and 3^rd^ dose |
|  | Partial adherence | Took 2^nd^ or 3^rd^ dose |

**Coding Framework – healthcare providers & managers**

| **Health system building blocks + context** | **Sub-node of health system building blocks +context** | **Definition to guide STOP-MiP2 health providers & managers coding**  **(🡪 study specific objectives)** |
| --- | --- | --- |
| Service delivery | Health facilities availability | How easy it is to access the health facilities, number and type of health facilities available for IPTp-DP  🡪 access to IPTp vs SST - Is IPTp more accessible because it does not require testing/can be given in the community without a lab?  🡪 Coverage of pregnant women who receive malaria prevention (IPTp and/or SST) |
|  | Services readiness | Availability of malaria prevention (i.e. IPTp-DP and SST) components (infrastructure, supplies, equipment, etc.) required to provide services. Also, how the intervention is perceived to fit with the existing system.  🡪 Did they feel prepared to implement IPTp DP (ie. training, supplies) |
|  | Health services provided | What malaria prevention services are provided? Including the perception of difficulty and challenges of current strategy  🡪 How IPTp is currently being delivered  🡪 Delivery and adherence to DOT  🡪 HP information to PW about dose 2 and 3 at home  🡪 Adherence to SST vs IPTp by gestation  🡪 Do they test for malaria before giving IPTp? |
|  | Strategies | What needs to be considered to have a smooth transition to IPTp-DP, includes possible challenges and adaptations to make it more palatable to PW.  🡪 community engagement strategies used  🡪 Potential alternative delivery strategies & adaptations |
|  | Quality of service | Including perceptions of patient satisfaction, and safety such as pharmacovigilance practice |
| Human resources | Context of current job | Information of the current job: position, role |
|  | Context of previous job | Information of the previous job: position, role |
|  | Staffing considerations | Are there an adequate number of trained staff? What happens when trained staff leave?  🡪 does IPTp change workload/additional tasks |
|  | Knowledge and training | Knowledge, skills, training, and perception of training received. Peer education and training requirements to do malaria prevention  🡪 is IPTp delivered according to the guidelines  🡪 What information is available to support HP in delivering IPTp DP; includes perceptions and availability of job aides |
|  | Attitude to malaria prevention | Perceptions of the advantages or disadvantages of using IPTp with DP vs the current policy of SST-DP includes perceptions of IPTp-DP vs SST-DP  🡪 benefits of IPTp (on malaria in pregnancy outcomes) |
|  | Supervision | Supervision of human resources and service delivery at the health facility level. Does it happen? Who does it? What does it involve? Do they have unanswered questions? |
| Health information systems | health information system being used | Anything related to the tools or registry developed/ updated/ changed with the current system in ANC, how they monitoring cases, adherence, commodity and supply chain at health facility  🡪 how and what information is used to make decisions about IPTp |
| Products and technologies | Supply chain and management | DP planning, storage, distribution and recording |
|  | Affordability | Affordability of providers to purchase DP and other commodities needed for the services  🡪 affordability of DP if women have to purchase outside facility |
|  | Risks/Side effects | The side effects of DP and its management  🡪 HP perception of risks/side effects  🡪 HP perceptions of how PW feel about side effects |
|  | Acceptability and Demand for DP | The demand of DP for IPTp vs treatment  🡪 HP acceptability towards DP for prevention  🡪 HP perceptions of PW acceptability to DP for prevention |
|  | Drug availability/stock outs | How do stockouts affect delivery?  🡪 Did women still accept to take IPTp after stocks resumed? |
| Health systems financing | Sources of financing | From what sources of financing to deliver IPTp-DP |
|  | Cost/ expenditure on health | Costs associated with implementing IPTp with DP, includes the cost and sustainability of IPTp-DP and available resources to meet the need. |
|  | Purchasing | How to purchase the services. Including, organizational Incentives & rewards. Extrinsic incentives such as goal-sharing awards, performance reviews, promotions, and raises in salary, and less tangible incentives such as increased stature or respect. |
| Leadership and governance | Health policies | Perceptions and access to the regulation, such as decree. Also, the policy required to implement the intervention. |
|  | Guidelines | Availability/access IPTp guidelines, provider comprehension of the guidelines |
|  | Participation or engagement | Who would need to be involved in implementing the new intervention at local and national levels |
|  | Ownership | Perception of key stakeholders about whether the IPTp-DP is externally or internally developed (source of intervention) |
|  | Supervision of the programme (higher level) | Perceptions of leadership in MOH vs TRF research team |
| Context | Structural Characteristics | Location, type of facility |
|  | Community Culture | Norms, values of community. Including what community do for malaria.  Perceptions of malaria burden in the community |
|  | Organizational culture | Norms, values of health facility or district health office |
|  | Political context | Political context in district level, including dynamic of local government leadership. |
|  | Health system context | General health system context |
