# Supplementary material for: Pregnant women’s acceptability of intermittent preventive treatment with dihydroartemisinin-piperaquine from user and provider’s perspectives: qualitative findings from the pilot implementation in Papua, Indonesia
Source: BMC Pregnancy Childbirth. 2026 Apr 6;26:536. doi: 10.1186/s12884-026-09036-x (PMC13188372; doi:10.1186/s12884-026-09036-x)
Supplement: Supplementary file 4 — Additional File 4. [file 12884_2026_9036_MOESM4_ESM.docx]

**Supplementary Table 1. Characteristics of health care workers at midline and endline**

| **Health providers** | **Midline (n=29)** | **Endline (n=40)** |
| --- | --- | --- |
| **Sex** |  |  |
| *Female* | 26 (90%) | 38 (95%) |
| *Male* | 3 (10%) | 2 (5%) |
| **Cadre** |  |  |
| *Nurse* | 10 (34%) | 10 (25%) |
| *Midwife* | 9 (31%) | 20 (50%) |
| *Pharmacist* | 10 (34%) | 9 (23%) |
| *In charge* | - | 1 (2%) |
| **Education** |  |  |
| *Diploma* | 16 (55%) | 20 (50%) |
| *Degree* | 12 (41%) | 18 (45%) |
| *Masters degree* | 1 (4%) | 2 (5%) |
| **Number of years work experience** |  |  |
| *2 years or less* | 4 (14%) | 1 (2%) |
| *Between 2 – 5 years* | 4 (14%) | 6 (15%) |
| *Between 5 – 8 years* | 12 (41%) | 18 (45%) |
| *More than 8 years* | 9 (31%) | 15 (38%) |
| **Length of time delivering IPTp-DP** |  |  |
| *6 months or less* | 29 (100%) | 5 (13%) |
| *7 to 12 months* | - | 11 (28%) |
| *> 13 months* | - | 24 (60%) |
